# Spinal cord stimulation for complex regional pain syndrome type 1 with dystonia: a case report and discussion of the literature

**DOI:** 10.12688/f1000research.3771.1

**Published:** 2014-04-30

**Authors:** Caroline Voet, Bernard le Polain de Waroux, Patrice Forget, Ronald Deumens, Etienne Masquelier

**Affiliations:** 1 Rehabilitation Medicine, Université Catholique de Louvain, Brussels, Belgium; 2 Multidisciplinary Reference Unit for Chronic Pain, Université Catholique de Louvain, Brussels, Belgium; 3 Anaesthesiology, Cliniques Universitaires Saint-Luc, Université Catholique de Louvain, Brussels, Belgium; 4 Institute of Neuroscience, Université Catholique de Louvain, Brussels, Belgium; 5 Neuropharmacology Unit, pole CEMO, Université Catholique de Louvain, Brussels, Belgium

## Abstract

**Background**: Complex Regional Pain Syndrome type 1 (CRPS-1) is a debilitating chronic pain disorder, the physiopathology of which can lead to dystonia associated with changes in the autonomic, central and peripheral nervous system. An interdisciplinary approach (pharmacological, interventional and psychological therapies in conjunction with a rehabilitation pathway) is central to progress towards pain reduction and restoration of function.

**Aim**: This case report aims to stimulate reflection and development of mechanism-based therapeutic strategies concerning CRPS associated with dystonia.

**Case description**: A 31 year old female CRPS-1 patient presented with dystonia of the right foot following ligamentoplasty for chronic ankle instability. She did not have a satisfactory response to the usual therapies. Multiple anesthetic blocks (popliteal, epidural and intrathecal) were not associated with significant anesthesia and analgesia. Mobilization of the foot by a physiotherapist was not possible. A multidisciplinary approach with psychological support, physiotherapy and spinal cord stimulation (SCS) brought pain relief, rehabilitation and improvement in the quality of life.

**Conclusion**: The present case report demonstrates the occurrence of multilevel (peripheral and central) pathological modifications in the nervous system of a CRPS-1 patient with dystonia. This conclusion is based on the patient’s pain being resistant to anesthetic blocks at different levels and the favourable, at least initially, response to SCS. The importance of the bio-psycho-social model is also suggested, permitting behavioural change.

## Introduction

Complex regional pain syndrome (CRPS) is a chronic pain disorder that usually affects the lower or upper extremities. Two types of CRPS have been described: CRPS type 1 (CRPS-1; reflex sympathetic dystrophy), normally triggered by a painful trauma without any detectable associated nerve lesion and CRPS type 2 (causalgia), involving a frank nerve injury. Both have similar symptomatology and are characterized by spontaneous pain that is disproportionate to the inciting event together with sensory, motor, autonomic and trophic changes ^1^. Its incidence in the Netherlands is estimated at 26.2 per 100,000 persons per year, with women being more frequently impaired than men ^2^. Physiopathogeny is complex, involving the central nervous system and peripheral neurogenic inflammatory processes ^3^. CRPS is a multifactorial disorder associated with an aberrant host response to tissue injury ^4^. Diagnostic criteria ^5^ rely on the clinical presentation. Specific additional diagnostic tests do not appear to be useful and often are expensive ^6^. CRPS is frequently associated with substantial disability, loss of quality of life and personal and societal economic burden ^4^. A combined pharmacological, interventional and psychological approach, in conjunction with a rehabilitation pathway, has been proposed for the management of CRPS. Pain reduction and restoration of function form the mainstay of therapy ^7^. Spinal cord stimulation (SCS) seems to be an effective and safe treatment of CRPS-1 ^8– 11^. Despite the diminishing effect of SCS over time, 95% of patients with an implant would be willing to repeat the treatment for the same result (if it had not already been implanted) ^12^. The success of SCS depends on the use of strict criteria for selecting patients that are likely to respond to this treatment ^8^. Unfortunately, necessary re-intervention as a result of technical problems with the implant are frequent, especially during the first two years following implantation ^9, 13^. Nevertheless, SCS seems to be cost-effective ^10, 12^.

We here report a case of severe CRPS in a 31-year-old woman, who did not respond to the usual treatments, including anesthetic blocks. SCS brought relief of pain, allowing rehabilitation.

## Case description

A 31-year-old white Caucasian woman suffering from repetitive ankle twisting underwent a second ligamentoplasty of the right ankle in 2008 for persisting instability. After intervention, she was treated with plaster-immobilization for seven weeks. Immediately after removal of the plaster, the patient reported intense pain and did not respond to standard painkillers (paracetamol, non-steroidal anti-inflammatory drugs and opioids), while physiotherapy became more and more difficult. Swelling of the right foot appeared, the scar re-opened and she presented with a dystonic posture of the right foot. Following an assessment by scintigraphy, possible CRPS was suspected. The diagnosis of CRPS-1 with dystonia was made and she was treated with several drugs, all of which were either without effect or poorly tolerated. A mobilization of the ankle was also performed under general anesthesia without long-lasting improvement.

In 2009, the patient was referred to a hospital where multidisciplinary management was started. Other treatments were tried, but without therapeutic benefits: dextropropoxyphene (600 mg/d), a buprenorphine patch (26.25 µg/h), hydromorphone (12 mg/d), clomipramine (50 mg/d), duloxetine (60 mg/d), pregabaline (300 mg/d), clonidine (0.30 mg/d), a lidocaine patch (2/day), intravenous pamidronate (30 mg/d) and methylprednisolone (64 mg/d). Three months later, the patient was referred to the Multidisciplinary Pain Center of CHU Mont-Godinne for administration of peridural and intrathecal anesthetic blocks (bupivacaine 5–15 mg). We noticed that the intrathecal block induced a contra-lateral motor block.

**Figure 1.**
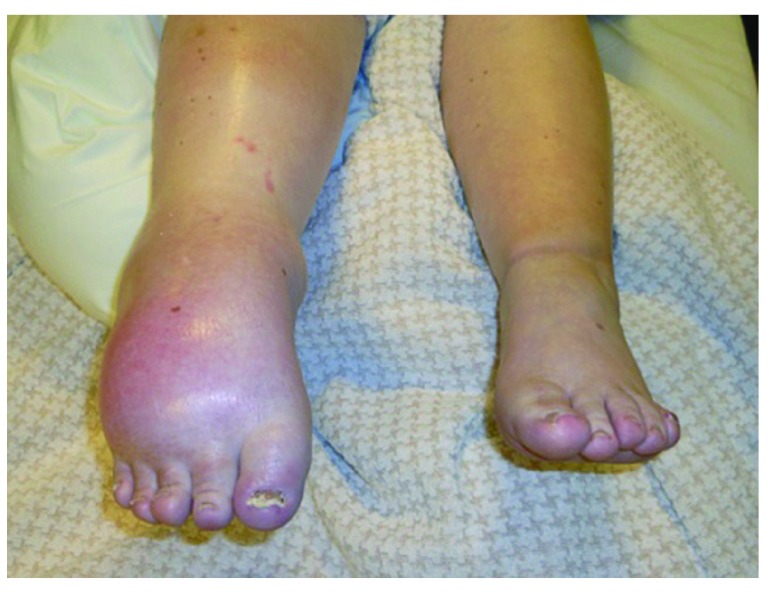
CRPS-1 (Complex regional pain syndrome): dystonic equinus of the right ankle, swollen foot and calf with tightened, pale, gleaming and cold skin.

When the patient was transferred to the Multidisciplinary Pain Center of the Cliniques Universitaires Saint-Luc in Brussels two months later, she presented with pain in the entire right foot, irradiating to just under the knee. The pain was permanent and reported on average at 8-9/10 on the VAS (Visual Analogue Scale). The patient described the pain as a burning feeling, which was enhanced by touch and mobilization. Walking required two canes, long distances necessitated a wheelchair. She also suffered from sleep and mood disturbances. Housekeeping was left to her husband and she took leave from her job at a child-care centre. Clinical examination showed a swollen foot and calf and tightened skin that was pale, gleaming and cold. The ankle was fixed in a dystonic equinus; only the toes presented active, though limited, movement of flexion-extension. The range of motion of the knee and hip were normal. Neurological assessment showed hypoesthesia like a sock on the right foot (up to the shin), hyperesthesia and hyperalgesia on the leg (on and above the knee- with allodynia (mechanical and dynamic rather than static). It was almost impossible for the patient to bear touch to the painful region, which meant that a complete sensory assessment could not be realized ( Figure 1). Evoked somesthetic potentials indicated normal lemniscal pathways coming from the lower limbs. The presence of a peripheral injury could not be evaluated because of the pain in the right leg.

In our Multidisciplinary Pain Center, the patient underwent the following algological techniques in order to try starting rehabilitation:

- Intravenous ketamine (6 mg/h, increasing to 20 mg/h): no effect and handicapping side-effects (nausea, drowsiness).
- Two anesthetic peripheral blocks of the right sciatic nerve at the knee, guided by echography (ropivacaine 115 mg, lidocaine 100 mg, clonidine 75 µg): no anesthesia and persistence of pain.
- Patches of clonidine (400 µg/day) and local anesthetic (lidocaine 5%) applied to the right foot: no effect.
- Anesthetic epidural block with a lateralized catheter. X-ray control with iohexol showed good epidural diffusion but injection of bupivacaine (25 mg) and lidocaine (100 mg) in combination with clonidine failed to produce any anesthesia or analgesia.
- Nine anesthetic intrathecal blocks using bupivacaine (5 to 13 mg), clonidine (60 to 75 µg) or baclofen (50 µg): no effect was found with 5 mg of bupivacaine, a partial positive effect lasting a maximum 2h 15min for 10 to 13 mg of bupivacaine as reported by the patient. Treatment by continuous infusion and bolus of clonidine and bupivacaine respectively had a partial effect but had to be interrupted because of post-lumbar-puncture-syndrome. Baclofen had no effect.
- Transcutaneous electrical nerve stimulation (TENS): the stimulations were not perceived below the knee, this treatment was thus ineffective.

Two months later, almost one year after onset of CRPS, the patient discontinued all analgesics because of lack of therapeutic benefits and side effects on her cognitive functions and personality. Subsequently, she start noticing an improvement in her mood and cognitive functioning. Pain and neuro-orthopedic status of the right leg remained the same. As she started to feel better mentally, she progressively broadened her activities and started spinning and hydrotherapy.

In 2010, almost one year later, SCS (epidural electrode, Medtronic ^®^, USA inserted percutaneously at lower lumbar level and pushed up to the T9 level) was trialled. During the testing period, we noticed a reduction in the intensity of hyperesthesia and allodynia of the right foot allowing touch and both active and passive mobilization of the foot, especially of the toes. Edema of the right foot decreased and vasodilation returned colouring the foot and warming it up. Importantly, the patient also experienced psychological relief. There was no effect on the equinus. After multidisciplinary discussion, it was decided to finally implant the pulse generator after about six weeks of testing.

**Figure 2.**
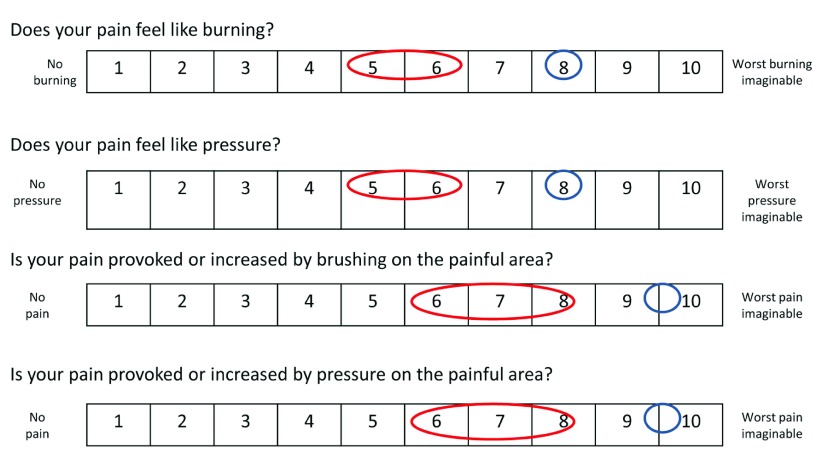
Neuropathic Pain Symptom Inventory (NPSI) evaluating the effect of Spinal Cord Stimulation (SCS) on neuropathic pain. In blue, the spontaneous sensation is indicated, without the SCS functioning. The values for neuropathic pain when SCS is on are shown in red, indicating a global reduction of pain.

**Figure 3.**
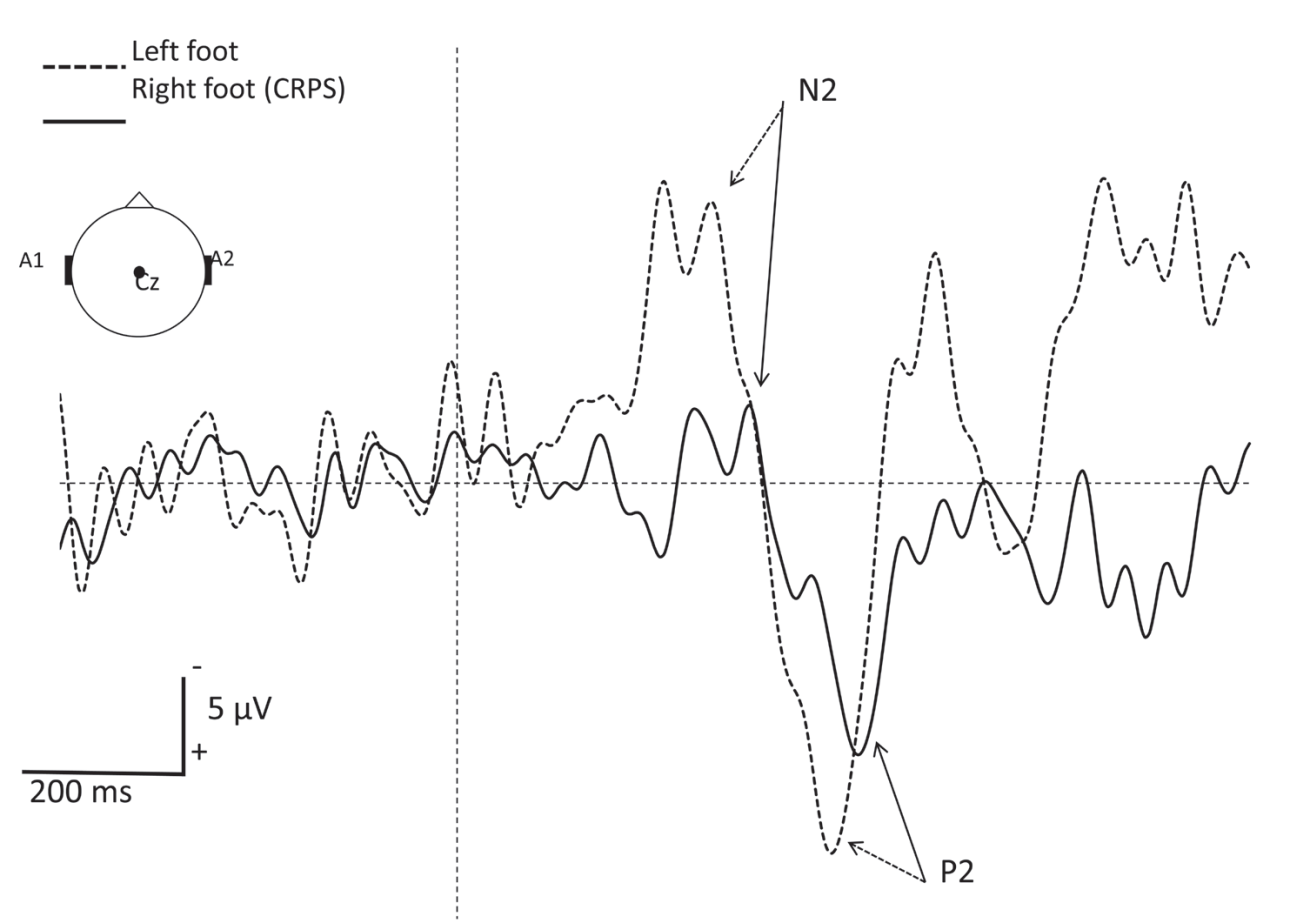
Grand average of A∂-fiber related laser evoked potentials (LEP) recorded at the vertex (Cz vs. A1-A2) after stimulation of the right (CRPS side) and left foot dorsum. Note the increased latency and reduced amplitude of LEP components in the affected foot as compared to the contralateral side. The vertical interrupted line represents the onset of the CO _2_ laser stimulus (duration 50 ms; surface area 79 mm ^2^; intensity 9.7 mJ/mm ^2^). Each side received 30 stimuli with an interstimulus interval of 8 to 15 s. The subject had to press a microswitch, held in her dominant hand, as fast as possible when perceiving the stimulus. To focus the patient’s attention, each stimulus was announced of 1.5 to 3 s beforehand, allowing her to fixe her gaze with open eyes during ±4 seconds to avoid eye movement artefacts.

At the time of writing this paper (2013), the patient is doing better. She has set up different strategies of coping, she is considering taking up her social life again and feels more optimistic about her future. Physiotherapy is now possible, but needs to be performed gently and progressively. Mobilization is performed during hydrotherapy. Orthopedic shoes have been manufactured in order to allow her to lean on her foot. Retrospectively, the Neuropathic Pain Symptom Inventory ^14^ (NPSI), used to determine the quality of pain relieved by SCS showed a partial relief of both spontaneous pain and evoked pain ( Figure 2). Laser evoked potentials (LEP) at one year after implantation of SCS showed a dysfunction of afferent small fibers (Aδ) from the right foot. No abnormalities where observed when stimulating the left foot and hand ( Figure 3).

## Discussion

About (9–49%) of patients with CRPS suffer from movement disorders, including loss of voluntary control, bradykinesia, dystonia, myoclonus and tremor. Dystonia occurs in approximately 20% of patients with CRPS and is characterized by fixed flexion postures of the fingers, wrist and feet that may vary in severity ^15^. The prevalence of movement disorders increases as the disease duration lengthens ^15^. The pathogenesis of CRPS and its relation to dystonia remain poorly understood. The central and peripheral nervous systems as well as immunological ^16^, psychological ^4, 15, 17, 18^ and genetic ^19^ factors seem to be implicated. We will only discuss neurological factors in this case report.

There is converging evidence for the role of the central nervous system in the physiopathogenesis of CRPS with dystonia. Central sensitization induced by tissue or nerve injury alters transmission and processing of peripheral sensorimotor input in the spinal cord. Associated with central disinhibition (both in the descending pathways and the brain itself), such changes set the stage for the development of movement disorders seen in CRPS ^15^. Cortical involvement in CRPS is suggested by mislocalizations of tactile stimuli, changes of size and organization of the somatosensory map, changes in motor cortex representation and body perception disturbances ^20^. The basal ganglia and parietal lobe seem especially related to some movement disorders such as dystonia and to hemineglect/inattention in CRPS ^21^.

Regarding the peripheral nervous system, large nerve fibers (proprioceptive afferents) do not seem to explain the underlying mechanisms of dystonia related to CRPS-1. Indeed, Van Rijn *et al.* ^22^ found no differences in somatosensory-evoked potentials (SSEP) in CRPS-1 patients with dystonia compared to healthy controls after spatio-temporal stimulation (confirming the integrity of “cortical proprioceptive afferent processing”). In relation to our case report, we would like to highlight the role of small nerve fibers in CRPS (C and Aδ). Pathological studies on chronic CRPS-1 limbs show degeneration of small (C and Aδ) nerve fibers which serve nociceptive and autonomic functions ^4^. However, this phenomenon does not seem to be specific to CRPS-1. Indeed, this degeneration is also seen in small-fiber-predominant polyneuropathies, which cause CRPS-like abnormalities ^23^. Oaklander ^23^ postulates that persistent CRPS-1 may represent a small-fiber-predominant mono- or oligoneuropathy that is initiated by a limb trauma. Moreover, dysfunction in small nerve fiber processing has been found by quantitative thermal testing in patients with CRPS-related dystonia ^24^. On the other hand, patients with pure small-fiber polyneuropathies never develop dystonia, implying that neither small-fiber dysfunction nor its central consequences are a driving force behind dystonia ^25^. Thus, it remains to be investigated whether nerve degeneration (i.e. dysfunction of small-diameter primary afferent nociceptor axons distal to trauma) causes CRPS-1 and/or dystonia ^4, 17, 24, 25^.

As such we do not have an unequivocal and clear explanation for the mechanism underlying dystonia in our CRPS-1 patient. However, LEP demonstrated dysfunction of small nerve fibers in the right foot is a possibility, as will be discussed in a later section of this article.

A recent alternative neurological hypothesis proposed by Ethier *et al.* ^26^, suggests a possible implication of the immune cells of the central nervous system, i.e. the microglia. These microglial could be activated in the brain as a result of a retrograde spread of neuroinflammation from the level of the spinal cord to the level of the motor cortex. As a consequence of microglial activation, functional changes may occur in the motor cortex. In predisposed individuals, these functional changes putatively trigger focal reduction of intracortical inhibition, a condition known to foster fixed dystonic postures. Further research in this area should help provide a better understanding of the mechanism underpinning CRPS-related dystonia.

Conventional therapies for the treatment of CRPS-1 with dystonia have poor efficacy. To our knowledge, there are no randomized controlled trials (RCT) of physical therapy, occupational therapy or pharmacotherapy in the treatment of movement disorders in CRPS ^27^. Strategies that enhance the central inhibitory state may benefit these patients ^15^. In some patients, the dystonia associated with CRPS responds markedly to intrathecal baclofen, a specific γ-aminobutyric acid (GABA) receptor agonist that inhibits sensory input to the neurons of the spinal cord ^28^. A rehabilitation program associating laterality recognition, mental imaging and mobilization in front of a mirror ^29^ is effective at reducing pain and increasing functioning by restoring sensory-motor integration ^6^. Most of these treatment options (including mirror visual feed-back) had been previously tried out by our patient, without delivering a satisfactory effect on pain relief or quality of life.

The same problem was noticed with interventional treatment (e.g. SCS). Over the last decade, only one other case of CRPS with resistance to local anesthetic blocks has been reported, involving a 12-year-old girl suffering from CRPS-1 of the right ankle ^30^. The mechanism of resistance to anesthetic blocks is currently unknown. The authors of this report, Maneksha *et al.* ^30^, proposed that changes in the dorsal horn cells of the spinal cord, secondary to activation of N-methyl-D-aspartate (NMDA) receptors, may play a role in the pathophysiology of this pain syndrome. Our case provides further support for the importance of neural changes associated with CRPS-1. The implication, in various pain conditions, of voltage-gated sodium channels, mainly isoforms Na(v)1.7 and Na(v)1.8, but also others of the nine isoforms (Na(v)1-9), has been well demonstrated. For example, overexpression of different isoforms of Na(v) (at least 1.7) is suspected to play a key role in the physiopathology of radicular pain, post-herpetic neuralgia and trigeminal neuralgia ^31^. In contrast, congenital deficiency of Na(v)1.7 is associated with inherited insensitivity to pain ^32^. In severe CRPS-1 patients, these receptors are clearly upregulated in keratinocytes ^33^. In the skin, their overexpression leads to neuronal hyperexcitability and pain, by increasing epidermal adenosine triphosphate (ATP) release and excessive activation of P2X receptors ^33^. Moreover, it has been shown that mutation of Na(v)1.7, which increases excitability of sensory neurons, can lead to a decrease of sympathetic activity when expressed on sympathetic neurons ^32^. A decrease in sympathetic activity, and a consequent increase of peripheral release of vasodilating peptides, leads to local changes such as erythema and edema, as seen in our patient. As a consequence, whether patients with CRPS-1 have an upregulation of Na(v) channels, at least Na(v)1.7, not only in keratinocytes, but also in the nervous system, may be worth investigating further.

Based on the inefficacy of potent Na(v) blockers (i.e. local anesthetics), we can indicate that major neurologic changes were present in our severe CRPS-1 patient. On the basis of the observed lack of response to local anaesthetics at different levels, we suspect that these changes were not only present in the periphery, but also in the (sciatic) nerve trunk, and in the spinal cord.

Despite her (relative) resistance to anesthetic blocks, our patient was a good responder to SCS. The mechanism of action of SCS is still incompletely understood and is frequently debated ( Table 1). The results of several studies, mostly on animal models but also in patients, suggest that the effect of SCS is to a large part mediated via GABA _B_ and muscarinic M _4_ receptors ^34– 37^. SCS induces GABA and acetylcholine (Ach) release in the spinal dorsal horn ^38– 40^ and activates descending serotoninergic pathways ^41^, all of which inhibit spinal nociception processing. In parallel, Truin *et al.* ^42^ demonstrated the role of NMDA receptors in the effect of SCS. Indeed, the combined use of SCS and sub-effective doses of intrathecal ketamine (an antagonist of NMDA receptors) resulted in a significant conversion of non-responders to SCS to responders to SCS. This effect could not be investigated in our patient as those treatments had not been given simultaneously. Besides, SCS is thought to affect peripheral vasodilatation via antidromic activation of spinal afferent neurons and inhibition of sympathetic efferents (small fibers). This effect seems to be mediated by calcitonin gene-related peptide (CGRP) and possibly nitric oxide (NO) ^43^.

**Table 1.** Different factors implicated in the mechanism of action of spinal cord stimulation. A brief review of the literature, comparing studies on animals and on humans.

Factor implicated	Reference	Animal studies	Human studies
NEUROPHYSIOLOGY GABA receptor (baclofen)	*Cui* J *et al.* (Neurosci Lett, 1998) ^34^ *Lind* G *et al.* (Eur J Pain, 2004) ^35^ *Lind* G *et al.* (Eur J Pain, 2008) ^36^ *Song* Z *et al.* (Neurosci Lett, 2008) ^37^	The effect of SCS could be enhanced by the corresponding agonists of GABA receptor and muscarinic receptor in animal models and in patients.	
Muscarinic receptor (Ach, oxotremorine)	*Cui* J *et al.* (Pain, 1997) ^38^ *Schechtman* G *et al.* (Pain, 2008) ^39^ *Schechtman* G *et al.* (Anesth Analg 2004) ^40^	SCS can induce GABA and Ach release, associated with diminished release of glutamate and aspartate in the spinal dorsal horn of animal models of neuropathic pain. The effect of SCS is to a large part mediated via GABA _B_ and muscarinic M _4_receptors.	N/A
GABA receptor (Serotonine)	*Song* Z *et al.* (Pain, 2009) ^41^	A rat model of mononeuropathy showed evidence that SCS activates the descending serotonergic pathways that may inhibit spinal nociceptive processing partially via GABAergic link.	N/A
NMDA receptor (antagonist Ketamine)	*Truin* M. (Eur J Pain, 2011) ^42^	N/A	The combined treatment of SCS and sub-effective doses of intrathecal ketamine in non-responders resulted in a significant reduction of the withdrawal threshold in all previous non- responders to SCS, thereby converting them into responders to SCS.
Spinal sympathic efferents (small fibers)	*Prager* J. (Pain Med, 2010) ^43^	Animal models of peripheral vasodilatation affected by SCS have shown the involvement of antidromic release of CGRP and possibly NO from small-diameter sensory neurons expressing the TRPV1 receptor. ERK may be an important signaling intermediary in this vasodilatary response to SCS and in animal models of neuropathic pain.	The involvement of sympathetic efferences in the vasodilatary response to SCS has been demonstrated in models of angina pectoris in patients.
CLINICAL EXAMINATION: Allodynia (mechanical)	*Smits* H *et al.* (Neuroscience, 2006) ^44^	N/A	The selection and subdivision of patient groups following the severity of mechanical allodynia may provide better pre-treatment prediction of possible therapeutic benefits of SCS.
	*Van Eijs* F *et al.* (Eur J Pain, 2010) ^13^		Brush-evoked allodynia may be a significant negative prognostic factor of SCS treatment outcome after one year in chronic CRPS-1.

GABA = γ-amino-butiric acid, Ach = Achetylcholine, NMDA = N-methyl-D-aspartic acid, CGRP = calcitonin gene-related peptide, NO = nitric oxide, TRPV1 = transient receptor potential vanilloïde 1, ERK = extracellular signal-regulated kinase.

Smits ^44^ suggests that the selection and subdivision by severity of mechanical allodynia may provide better pre-treatment predictions of the possible therapeutic benefits of SCS. These results match with those of Van Eijs ^13^ suggesting that brush-evoked allodynia may be a significant negative prognostic factor of SCS treatment outcome after one year in chronic CRPS-1. The chances of achieving and maintaining successful pain reduction drop from 81% to 31% if allodynia is present ^13^. However this notion partially conflicts with our observations. In our patient allodynia evoked by brushing or pressure on the painful area was present before SCS was tested and evoked pain scores were partially relieved by SCS, according to the NPSI ( Figure 2).

It is important to emphasize that the reason someone with chronic pain gets better has as much to do with the nonspecific effects of treatment as with the treatment itself ^45^. For instance, why did most of the invasive techniques undergone by our patient have no effect? And why was modulation of pain by SCS possible later on? Much of this has to do with the particular aspects of the human brain and the individual’s need to interpret pain ^46^. Among those nonspecific treatment effects, factors that can impact on pain perception include patient-specific factors (degree of anxiety, desire to get better; improved coping etc.) and the interpersonal relationship between that person and their physician (perception of attention and caring ^47^; major value of education, reassurance and counseling ^48^; heightened expectations ^49^). In the context of chronic pain patients who are challenging to treat, the importance of communication style is paramount ^50^. It is important to remember that empathy, mutual respect and an open doctor-patient collaboration in the treatment are excellent skills in the interaction with patients ^51^. Jamison ^45^ suggests that the therapeutic quality of the practitioner’s manner and the role of the patient’s expectations of treatment are very powerful. We need to maximize those nonspecific effects of care in reducing the suffering of individuals with pain.

This case suggests the importance of behavioral change as part of the treatment of CRPS. First, the patient expected an explanation of her illness based on the bio-medical model ^52^. This concept postulates that disease is fully accounted for by deviations from the norm of measurable biological (somatic) variables. A complex phenomenon is ultimately derived from a single primary principle (reductionism) and the mental component is separated from the somatic component (dualism, specifically psychophysic parallelism). Progressively, helped by a psychosocial accompaniment conducted by a clinical psychologist, her conception of illness converted into a bio-psycho-social model, accounting for human experiences besides the somatic abnormalities. That psychological work seemed to be necessary before trying out the SCS, even if, in this case, it remains, at least partially, hypothetical. Nevertheless, we propose that the bio-psycho-social model of rehabilitation in the treatment of CRPS would be an important step for achieving a change of behavior to acceptation of a disabling situation.

Finally, the LEP realized on our patient suggest pathology of the small fibers (C and Aδ). Whether this is the cause or the result of CRPS is uncertain. To our knowledge, no article has been published exploring the link between LEP results and the outcome of treatment by SCS in CRPS-patients. This is an interesting topic for future research.

## Conclusion

The resistance of pain to anesthetic blocks at different levels of the nervous system and the favorable response to SCS emphasizes the complexity of the pathophysiology of CRPS associated with dystonia in our case. This case is also notable because of its complex presentation: initial resistance to several treatments with subsequent reduction of pain by SCS. This poses the interesting question of the role of nonspecific treatment effects. Finally, it illustrates the importance of the bio-psycho-social model.

## Consent

Consent for the publication of clinical details and images was obtained from the patient.
