# Supplementary material for: Spinal cord stimulation for complex regional pain syndrome type 1 with dystonia: a case report and discussion of the literature
Source: F1000Res. 2014 Apr 30;3:97. [Version 1] doi: 10.12688/f1000research.3771.1 (PMC4168752; doi:10.12688/f1000research.3771.1)
Supplement: Peer review history

# Peer review: Referee response for version 1

**DOI:** 10.5256/f1000research.4040.r5717

**Published:** 2014-09-09

**Authors:** Michael Erdek

**Affiliations:** 1 Department of Anesthesiology & Critical Care Medicine, John Hopkins University, Baltimore, USA

There are two comments I have regarding the submission:

In the case description, the authors mention mobilization of the foot by a physiotherapist was not possible. Can they be more specific as to why that was the case?

In the Discussion, you mention that 9-49% of CRPS patients suffer from movement disorders. What is your source for that figure?

I have read this submission. I believe that I have an appropriate level of expertise to confirm that it is of an acceptable scientific standard.

# Peer review: Referee response for version 1

**DOI:** 10.5256/f1000research.4040.r5167

**Published:** 2014-07-22

**Authors:** Frank van Eijs

**Affiliations:** 1 Department of Anaesthesiology, Sint Elisabeth Hospital, Tilburg, Netherlands

The authors describe an interesting but not infrequently occurring case of therapy resistant CRPS. The report is well described. Only few issues need to be addressed. **Introduction**

- " *involving a frank nerve injury*" may be replaced by " *involving a detectable large fiber nerve injury*"

**Case description**

- *“in 2008..”* add the month
- *“In 2009...”* add the month
- " *the patient reported intense pain and did not respond to*"... replace and with which
  “ *Subsequently she start noticing...”* replace start with started

**Discussion**

- Explain P2X receptors
- *“It is important to emphasize that the reason* why *someone with chronic pain gets better...”*
- *“factors that can impact...”* replace can with may.

In conclusion: although it's scientific value is of minor importance because of the nature of the article (being a case report), the report certainly is worth indexing.

F. van Eijs, MD, PhD

I have read this submission. I believe that I have an appropriate level of expertise to confirm that it is of an acceptable scientific standard.
